# Molecular Mechanisms of Action of Herbal Antifungal Alkaloid Berberine, in *Candida albicans*

**DOI:** 10.1371/journal.pone.0104554

**Published:** 2014-08-08

**Authors:** Sanjiveeni Dhamgaye, Frédéric Devaux, Patrick Vandeputte, Nitesh Kumar Khandelwal, Dominique Sanglard, Gauranga Mukhopadhyay, Rajendra Prasad

**Affiliations:** 1 School of Life Sciences, Jawaharlal Nehru University, New Delhi, India; 2 Special Centre for Molecular Medicine, Jawaharlal Nehru University, New Delhi, India; 3 Sorbonne Universités, UPMC Univ Paris 06, UMR 7238, Laboratoire de genomique des microorganisms, Paris, France; 4 Institute of Microbiology, University of Lausanne and University Hospital Center, Lausanne, Switzerland; New Jersey Medical School, Rutgers University, United States of America

## Abstract

*Candida albicans* causes superficial to systemic infections in immuno-compromised individuals. The concomitant use of fungistatic drugs and the lack of cidal drugs frequently result in strains that could withstand commonly used antifungals, and display multidrug resistance (MDR). In search of novel fungicidals, in this study, we have explored a plant alkaloid berberine (BER) for its antifungal potential. For this, we screened an in-house transcription factor (TF) mutant library of *C. albicans* strains towards their susceptibility to BER. Our screen of TF mutant strains identified a heat shock factor (*HSF1*), which has a central role in thermal adaptation, to be most responsive to BER treatment. Interestingly, *HSF1* mutant was not only highly susceptible to BER but also displayed collateral susceptibility towards drugs targeting cell wall (CW) and ergosterol biosynthesis. Notably, BER treatment alone could affect the CW integrity as was evident from the growth retardation of MAP kinase and calcineurin pathway null mutant strains and transmission electron microscopy. However, unlike BER, *HSF1* effect on CW appeared to be independent of MAP kinase and Calcineurin pathway genes. Additionally, unlike hsf1 null strain, BER treatment of Candida cells resulted in dysfunctional mitochondria, which was evident from its slow growth in non-fermentative carbon source and poor labeling with mitochondrial membrane potential sensitive probe. This phenotype was reinforced with an enhanced ROS levels coinciding with the up-regulated oxidative stress genes in BER-treated cells. Together, our study not only describes the molecular mechanism of BER fungicidal activity but also unravels a new role of evolutionary conserved *HSF1*, in MDR of Candida.

## Introduction

*Candida albicans* is a part of normal commensal flora of human body, which becomes one of the most resilient fungal pathogen under low host immunity [1], [2]. Available antifungals predominantly include azoles, echinocandins, polyenes and allylamines [3]. Each of these classes of antifungals have a distinct mode of action, for example azoles target heme protein, cytochrome P450 lanosterol 14 α demethylase thereby impeding conversion of lanosterol to fecosterol and subsequently blocking ergosterol biosynthesis [4]. Echinocandins interfere with CW synthesis by inhibiting β1-3 glucan synthase [5]. Polyenes have affinity to bind membrane sterols that results in the formation of aqueous pores ensuing the leakage of crucial cellular components and subsequent cell death [6]. Allylamines are relatively newer class of antifungal that also inhibit ergosterol biosynthesis but by specifically targeting squalene epoxidase [7].

Notwithstanding the fact that some pathogenic Candida species are intrinsically resistant to antifungals, prolonged use of drugs also leads to an emergence of multidrug resistant (MDR) strains posing additional clinical challenges [8]. Because of the limited arsenal of antifungals, there is constant need to find new antifungals with novel targets. In this context, herbal antifungals have acquired significance owing to their natural origin [9]–[11]. Besides herbal compounds there are innumerable non-herbal chemical compounds with antifungal potentials. For example, gentian violet has been used to treat recurrent vulvovaginal candidosis [12]. Idarubicin, a DNA topoisomerase inhibitor is effective against *Aspergillus niger*, *Cryptoccocus neoformans* and *C. glabrata* [13], caffeine exhibits antifungal potential by interfering with MAPK signaling and CW integrity pathway [14]. Recently, our group has shown that a bacteriological stain malachite green (MG) exerts antifungal effect by shifting the metabolic flux of Candida cells towards fermentation, increasing generation of ROS and leading to cell necrosis [15].

Isoquinoline alkaloid berberine (BER), which is bright yellow colored, is widely used for dyeing wool, leather and wood. BER is shown to elicit anticancerous, antidiabetic, anti atherosclerotic and neuroprotective properties. Additionally, BER also exhibits antibacterial, and has also shown anti-inflammatory potentials [16], [17]. Although there are instances where BER is shown to elicit antifungal activity against *C. albicans* and *C. glabrata* [18], how it exerts its effect is not understood.

In the present study, we attempted to elucidate the antifungal effect of BER on *C. albicans* and non-*albicans* species. For this, we screened the transcriptional factor (TF) mutants in house library of *C. albicans* and identified a mutant of heat shock factor (*HSF1*) that was hypersusceptible to BER [19]. *HSF1* activates transcription via heat shock element (HSE) in response to thermal adaptation. Our study provides evidence that the treatment of Candida cells with BER compromises CW integrity via the calcineurin pathway leading to cell death. The study also assigns a new role to *HSF1* in combating MDR.

## Material and Methods

### Strains and Media

All the media components were purchased from HiMedia (Mumbai, India). BER, 2′, 7′ -dichlorofluorescein diacetate (DCFDA), and glycerol were purchased from Sigma chemicals Co. (St. Louis, MO). Ascorbic acid (AA) was purchased from SRL (Mumbai, India). Ranbaxy, India generously provided FLC. AMB, CAS, CR, CFW, SDS, TRB were purchased from Sigma chemicals Co. (St. Louis, MO) Superscript-II Reverse transcriptase enzyme, oligo dt_(18)_primer and random primer from Invitrogen, USA. dATP, dGTP, dTTP and dCTP were bought from Sigma. All the yeast strains were grown in yeast-extract peptone dextrose (YPD) agar and incubated at 30°C. All the strains were stored as 15% glycerol stocks in −80°C storage which were freshly revived on YPD before beginning of the experiment. Details of all the strains used in the study is described in table S1 in File S1.

### Growth curve and serial dilution assays

Freshly revived *C. albicans* strains were inoculated at 0.1 OD_600_ in YPD in four different tubes. BER was added at 100, 150, 200 µg/ml in three of the tubes while no BER was added to the control tube. Cells were allowed to grow at 30°C at 200 rpm. 1 ml of culture is withdrawn from each tube at interval of 4 hours and absorbance is taken at 600 nm. Serial dilution assays were done in both liquid and solid YPD medium for testing the drug susceptibility essentially by following the guidelines provided by CLSI (Clinical and Laboratory Standards Institute) [20],[21]. Concentration of each drug used in the study is provided in parenthesis BER (200 µg/ml, 300 µg/ml depending on the WT strain used), doxycyclin (DOX; 0.6 µg/ml), caspofugin (*C*A*S*; .05 µg/ml), amphotericin B (AMB; 4 µg/ml), calcofluor white (CFW; 60 µg/ml), congo red (CR; 150 µg/ml), SDS (0.05%), ascorbic acid (AA; 0.3 µg/ml).

### Screening of TF mutant library for BER susceptibility

We have employed an in-house TF mutant library containing 240 homozygous mutant strains individually deleted for genes with DBD (DNA binding domain) by UAU transposition (∼70%) or by URA blaster technique or by PCR product recombination PCR [15]. The TF mutants were subjected to the screening for BER susceptibility using serial dilution assays as described above. This was done thrice and the TF mutant strain that was reproducibly susceptible to BER was tested in the final screen.

### End point comparative reverse transcription PCR (RTPCR)

RNA was extracted using RNeasy mini kit Qiagen essentially by following their protocol. RNA is quantitated by NanoDrop 2000 UV-Vis spectrophotometer from Thermo Scientific. 1 µg/ml of DNase treated RNA was taken further for cDNA preparation using RevertAid H Minus Reverse Transcriptase kit from Thermo Scientific using protocol provided by the manufacturer. 1 µl of the cDNA product was used for amplification using Taq DNA polymerase from New England Biolabs in a total reaction volume of 50 µl. Primers used for the amplification of target genes are listed in table S2 in File S1.

### Transmission electron microscopy (TEM)

TEM was performed to examine the effect of BER on the cell wall of *HSF1tet/hsf1Δ* strain in comparison with the WT as described elsewhere [22]. Cells were grown to mid-exponential phase and treated with DOX, BER or BER +DOX. The cells were washed with PBS buffer to remove the media and then cells were chemically fixed using 2% glutaraldehyde at 4°C for 2 hours followed by 0.5% osmium tetroxide. The cells were finally embedded in epon resin. The samples were visualized by TE microscope by Jeol JEM-2100F, Tokyo Japan.

### BER transport

BER transport inside the cells was measured exploiting its fluorescence. BER has excitation wavelength of 405 nm and emission is at 520 nm [23]. Late stage cells were washed with PBS twice and the cells were resuspended in PBS to 0.1 OD_600_. Cells were incubated for 1 hour and then washed cells were resuspended in PBS with 2% glucose with BER (MIC_50_ concentration). Fluorescence detection in cells was carried out with a Fluorescence Activated Cell Sorter (FACS) caliber flow cytometer (Becton-Dickinson Immunocytometry Systems, San Jose, CA) at FL-1. Ten thousand events were counted and fluorescence intensity was compared between untreated cells and BER-treated cells using Cell Quest software.

### Measurement of ROS (Reactive Oxygen Species) generation

Endogenous ROS generation was measured using DCFDA (2′,7′-Dichlorofluorescin diacetate) which is an oxidant sensitive fluorescent probe [24]. The method employed is previously described in details [15]. Briefly, wild type cells were inoculated in YPD broth at 0.1 OD_600_ and allowed to incubate at 30°C until the OD_600_ reaches 0.8. BER (MIC_50_) was added to the cells and same volume of mock in the control then allowed to grow further for 80 min. We used antioxidant AA for reversal of generation of ROS. Cells were pretreated with 5 mM AA 60–90 min and then incubated in presence of BER for 80 mins after washing the cells, 10 µM DCFDA was added. Cells were then kept at 30°C for 30 min. Fluorescence was measured (Excitation and emission at 488 nm and 540 nm respectively) and quantified by FACS as mentioned above.

### Evaluation of induction of apoptosis by BER treatment

Cells undergoing apoptosis was determined by the externalization of PS (phosphatidylserine), an apoptotic marker, by using FITC (Fluorescein isothiocyanate) labeled Annexin V kit (FITC Annexin V Apoptosis Detection Kit I, BD Biosciences). Exponentially growing cells were treated with BER (at MIC_80_; 200 µg/ml). Cells were then treated with zymolyase to obtain spheroplasts, which were incubated with FITC Annexin V and PI (propidium iodide). The fluorescent signal was assessed using a Fluorescence Activated Cell Sorter (FACS) caliber flow cytometer (Becton-Dickinson Immunocytometry Systems, San Jose, CA). We used 488 nm excitation and a 515 nm band pass filter for FITC detection and a filter >560 nm for PI detection. A total of 10,000 events were counted at the flow rate. Data analysis was performed using Cell Quest software. Cells labeled with FITC indicate onset of apoptosis while those with PI label predominantly show the presence of necrotic cells (AnnV/PI) [15].

### Determination of active mitochondria using MitoTracker

Exponentially growing cells were treated with BER (MIC_50_), for 80 min washed and resuspended in YPD and treated with 200 nM of MitoTracker Deep Red FM (MTR-FM) [25]. The cells were allowed to incubate for 30–45 min at 30°C with 200 rpm. The cells were washed and resuspended in 400 µL of PBS fluorescence is measured by FACS analysis as described above. MTR-FM has excitation and emission spectra of 644 nm and 665 nm respectively.

## Results

### Antifungal potential of BER does not depend on major efflux pump proteins

**Figure 1:**
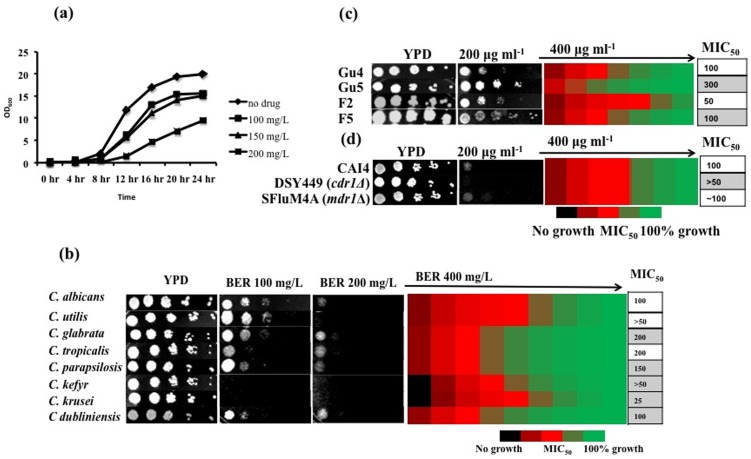
Antifungal potential of BER (a) Growth curve of WT *C. albicans* cells at 100, 150 and 200 µg/ml, (b) serial dilution assays in solid (left panel) and liquid medium for testing BER susceptibility of *C. albicans* and non albicans species. (c) Serial dilution assays of *CDR1* (Gu5) and *MDR1* (F5) overexpressing and (d) their deletions strains in presence of BER.

For an in depth analysis of mechanism of action of antifungal potentials of an herbal alkaloid BER, we have examined the growth pattern of *C. albicans* and observed that BER maximally retarded cell growth at 200 µg/ml (Figure 1(a)). In serial dilution assays, BER at 200 µg/ml was also effective against all the tested non-*albicans* species with variable susceptibilities (Figure 1(b)). For instance, *C. utilis*, *C. kefyr* and *C. krusei* were relatively hypersusceptible to BER and their MIC_50_ ranged between 25 µg/ml and >50 µg/ml, while *C. glabrata* and *C. tropicalis* were the least susceptible species with MIC_50_ of 200 µg/ml in contrast to *C. albicans*. Notably, BER has reported to be non-toxic to human cells [26].

One of the predominant mechanisms to cope up with the antifungal stress is an overexpression of drug efflux pump encoding genes such as *CDR1* and *MDR1* belonging to the ABC (ATP Binding Cassette) and MFS (Major Facilitators) super families of transporters, respectively [27]. We assessed the implication of these pump proteins in withstanding BER stress. For this, we used azole-susceptible (AS) and azole-resistant (AR) clinical isolates overexpressing these pump encoding genes [28], [29], and examined the effect of BER. As depicted in Figure 1(c) and 1(d), the susceptibility to BER in *CDR1* or *MDR1* overexpressing strains was only modestly affected as compared with susceptible strains. The possibility that the observed modest increase in susceptibility of clinical resistant isolates towards tested drugs might arise due to acquired mutations in other genes, that can also not be ruled out. To check the specific roles of these transporters, we have used strains that were individually disrupted for these genes but the susceptibility in these nulls (DSY449 and SFluM4A) remained unaffected in comparison to the respective WT strains. Together, these results imply that efflux pumps do not impact BER activity (Figure 1 (c) and 1(d)).

### *HSF1* levels affect susceptibility to BER and collateral susceptibilities to other drugs

**Figure 2:**
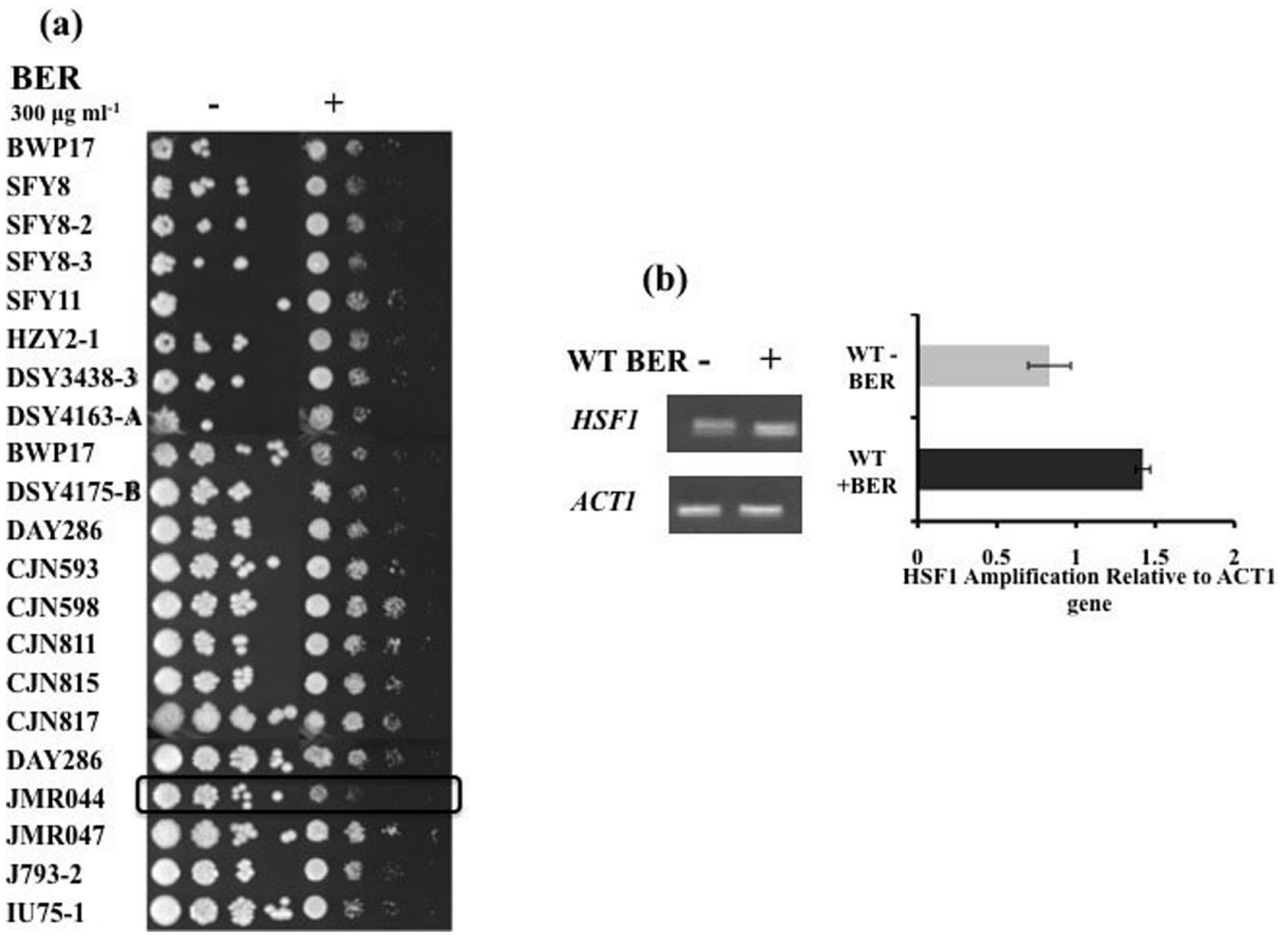
TF mutant library screening (a) Serial dilution assays of TF mutant strains in the presence of BER, (b) end point comparative RTPCR of *HSF1* (gene deleted in JMR044) in WT strain (DAY286) in presence and absence of BER.

To understand the mechanism behind susceptibility to BER, we carried out a screen of an in-house library of TF mutant strains of *C. albicans* [15]. There are ∼275 genes that contains DBD in *C. albicans* these genes were deleted individually as explained in materials and methods section. All the strains that appear reproducibly (in triplicate) susceptible were subjected to a final screen as shown in Figure 2(a). Among all the TFs, *HSF1*, a heat shock factor mutant (JMR044) was most susceptible to BER treatment (MIC_50_ 25 µg/ml) in comparison with WT strain (DAY286) (MIC_50_ 100 µg/ml) (figure S1 in File S1). End point comparative RTPCR revealed a slight increase in the expression of *HSF1* after BER treatment in *C. albicans*, thus implying a direct effect of BER on its expression (Figure 2(b)).

**Figure 3:**
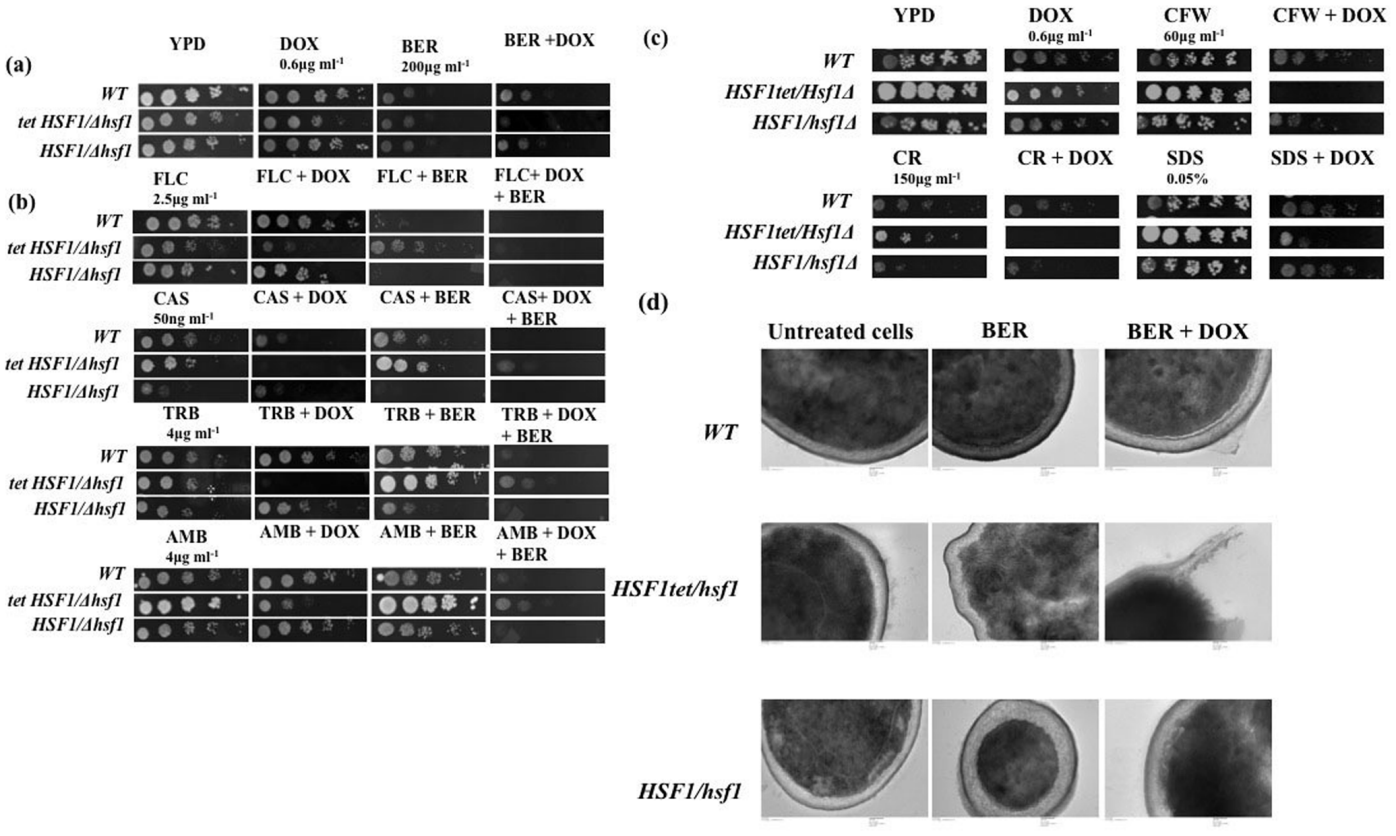
*HSF1* conditional mutant is susceptible to various antifungal drugs (a) susceptibility WT, *HSF1* conditional mutant and *HSF1* heterozygous for BER (b) different classes of antifungal drugs; FLC, CAS, TRB, AMB, and their combination with BER, (c) CW perturbing agents; CFW, CR, SDS (d) TEM images of WT, *HSF1* conditional mutant and *HSF1* heterozygous in presence of BER.

*HSF1* is an essential gene and it could be likely that this TF mutant that was generated by UAU transposition could still exhibit *HSF1* activity due to the chosen disruption method [19], [30]. To reconfirm *HSF1* impact on MDR, we employed a conditional *HSF1* mutant (*hsf1Δ/tet-HSF1*) in which *HSF1* expression is under control of the TET promoter [19]. We tested the susceptibility of the *HSF1* conditional mutant in presence of TET analogue, DOX, which shuts off the expression of *HSF1*. The results mirrored the BER susceptibility phenotype that was observed with the mutant strain JMR044 of TF library screen (Figure 3(a)). We also verified whether conditional mutant *hsf1Δ/tet-HSF1* was collaterally affecting susceptibility to other drugs. FLC, AMB, TRB, and CAS (caspofungin). Additionally, we have also evaluated the effect of combination of BER and these drugs on conditional mutant *hsf1Δ/tet-HSF1*. Interestingly, the conditional *HSF1* mutant was highly susceptible towards different classes of antifungal drugs including FLC, AMB, TRB, and CAS (caspofungin) (Figure 3(b)). Our susceptibility assays revealed that *hsf1Δ/tet-HSF1* not only displayed enhanced susceptibility to BER but also to drugs acting on membrane ergosterol (TRB, FLC, AMB) or inhibit CW synthesis (CAS) (Figure 3(b)) [31], [32]. However, combination of BER and above mentioned drugs did not seem to enhance the susceptibility of *HSF1* conditional mutant (Figure 3(b)). This highlights that the effects of BER and *HSF1* leading to drug susceptibility are manifested by independent circuitry. The impact of *HSF1* on drug susceptibilities appeared to be independent of known attributes of MDR [33], [34]. For example, the expression of *CDR1*, *CDR2*, *MDR1*, *ERG11*, *TAC1*, *UPC2* did not change in the *HSF1* conditional mutant in presence and absence of BER (figure S2 in File S1).

Susceptibility of *hsf1Δ/tet-HSF1* towards ergosterol and CW synthesis inhibitors led us to evaluate specific effect of CW perturbing agents such as SDS (sodium dodecyl sulphate), CR (congo red) and CFW (calcofluor white) [35] on this mutant. We performed serial dilution assays in the presence of these CW perturbing agents. As evident from Figure 3(c), *hsf1Δ/tet-HSF1* was susceptible to SDS (0.05%), CR (150 µg/ml) and CFW (60 µg/ml). The impact of *HSF1* on CW was reinforced by TEM of *hsf1Δ/tet-HSF1* strains. One could visualize in TEM pictures, the damage to the CW of *hsf1Δ/tet-HSF1* strains. When *hsf1Δ/tet-HSF1* strains were treated with BER, the impact appeared to be more aggravated as was evident from the rupturing of the CW and shrinkage of the cytoplasm of *hsf1Δ/HSF1* cells after BER treatment (Figure 3(d)). Interestingly, the damage to CW as revealed by TEM picture analyses following BER treatment was not as severe as compared to untreated *hsf1Δ/HSF1* cells (Figure 3(d)).

### BER affects CW integrity independent of *HSF1*

**Figure 4:**
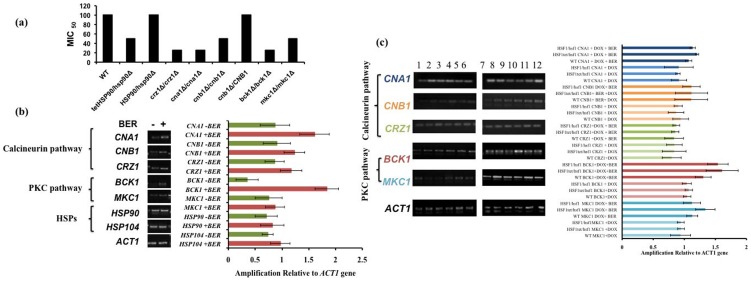
Effect of BER on CW integrity mutants (a) serial dilution assay of calcineurin and MAP kinase pathway and *HSP90* gene deleted to evaluate BER MIC_50_, (b) end point comparative RTPCR of genes involved in CW integrity in WT *C. albicans* cells in presence and absence of BER, (c) and in *HSF1* conditional mutant lane indicates 1: WT, 2: HSF1 TET/hsf1, 3: HSF1/hsf1, 4,5,6,: +Doxy, 7,8,9 :+BER, 10, 11, 12: +Doxy+Ber.

Taking clue from *hsf1Δ/tet-HSF1*, which was susceptible to CW inhibitors, we explored whether the genes involved in the CW integrity pathway were affected in BER-treated cells [36]. By employing liquid assay according to the CLSI protocol, we compared BER MICs in mutant strains for the genes involved in heat shock (*HSP90*), calcineurin (*CNA1*, *CNA2*, *CRZ1*), and MAP kinase pathways (*BCK1*, *MKC1*). As evident from the assay, calcineurin (*crz1Δ/crz1Δ*, *cna1Δ/cna1Δ*) and MAP kinase pathways (*bck1Δ/bck1Δ*) mutants were more susceptible to BER treatment with MIC_50_ of 25 µg/ml as compared to wild type (100 µg/ml) (Figure 4(a)). End point comparative RTPCR confirmed that the expression of genes involved in these pathways (including *CRZ1*, *CNA1* and *BCK1*) was increased upon BER treatment (Figure 4(b)). Notably, depletion of *HSF1* levels did not arrest the increased expression of these genes upon BER treatment (Figure 4(c)) in combination of DOX indicating BER effect on these genes is independent of *HSF1*.

### BER treatment results in mitochondrial dysfunction

**Figure 5:**
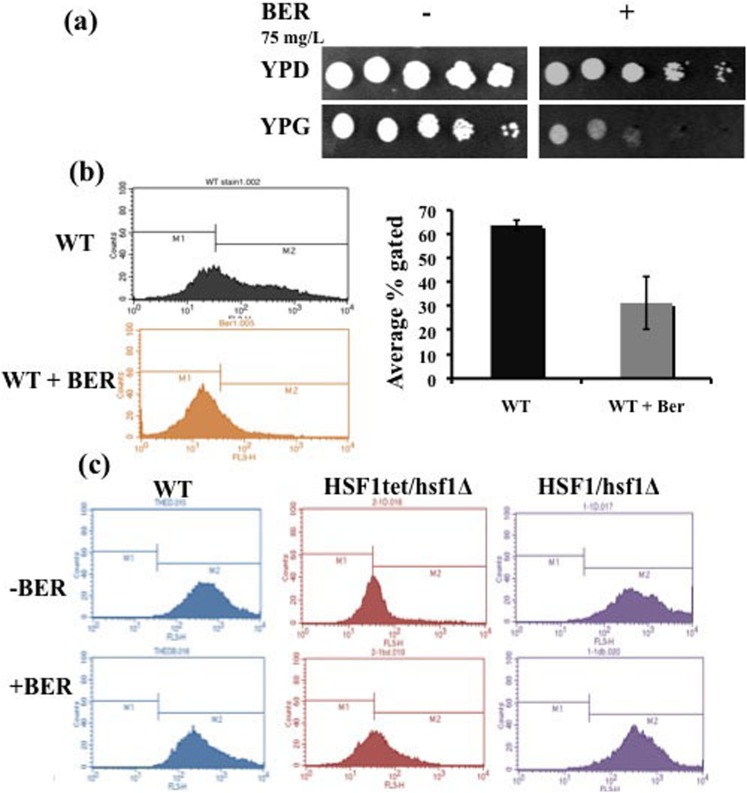
BER treatment results in dysfunctional mitochondria (a) growth of *C. albicans* cells in non-fermentable carbon source (glycerol) in presence of BER (b) MTR labeling of the active mitochondria by FACS in *C. albicans* WT cells in presence and absence of BER, bar graph representing number of events gated (c) MTR labeling were also done in WT, *HSF1* conditional mutant and *HSF1* heterozygous strains in presence and absence of BER.

To assess functional mitochondria, we compared the growth of BER treated cells in non-fermentative and in fermentative carbon sources and compared it with the growth of untreated cells. We observed that, even at non-lethal BER concentration (below MIC_50_; 75 µg/ml), cells were unable to grow when non-fermentable glycerol was provided as sole carbon source (Figure 5(a)). We addressed the mitochondrial activity by employing MTR-FM probe [25]. MTR-FM probe is known to passively diffuse across yeast cell membrane and to accumulate in active mitochondria, which can be visualized by enhanced fluorescence. Using flow cytometry depicted in Figure 5(b) BER-treated cells showed poor fluorescence intensity as compared to untreated cells. Together, poor growth in non-fermentable carbon source and decrease in MTR-FM fluorescence reinforced dysfunctional mitochondria following BER treatment. Notably, *HSF1* conditional mutant did not show any impact on mitochondrial functional status (Figure 5 (c))

### Determination of generation of ROS and apoptosis on BER treatment

**Figure 6:**
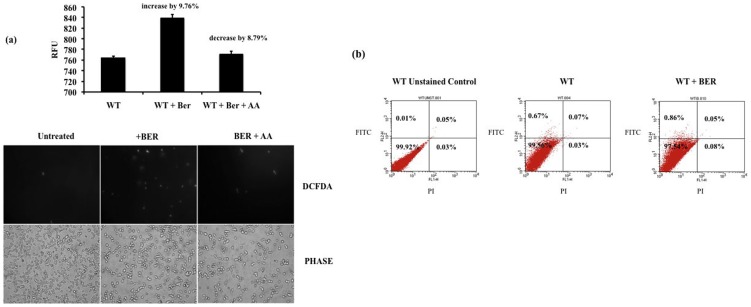
Determination of endogenous ROS generation by BER and induction of apoptosis (a) (upper panel) bar graph representing relative fluorescent units when cells were treated with DCFDA in presence and absence of BER, AA is added to revert the ROS production, (lower panel) fluorescent microscopy images of WT *C. albicans* cells labeled with DCFDA, (b) Cytometric determination FITC Annexin V labeling in WT cells treated with BER.

Mitochondrial dysfunction on BER treatment led us to determine the status of ROS in presence of BER. To measure the changes in ROS generation we exploited a fluorescent molecule chloromethyl-dichlorodihydrofluorescein diacetate (CM-H_2_DCFDA), which can readily enter into the cells and is sensitive to redox changes [24]. It is deacetylated by endogenous esterase into dichlorofluorescein, which fluoresces on oxidation by ROS. Wild type cells treated with a MIC_50_ concentration of BER (100 µg/ml) were allowed to incubate in presence of 10 µM of CM-H_2_DCFDA. As shown in Figure 6, fluorescence increased after BER treatment indicating generation ROS. The increase in ROS following BER treatment could be partially reversed by the addition of antioxidant such as AA [37]. This implied that BER treatment results in production of ROS, which could contribute to the antifungal effect exerted by BER.

To estimate whether generation of ROS leads to apoptosis on BER treatment, we checked for externalization of phosphatidyl serine (PS), a marker for apoptotic cells. Annexin V (Annexin V Apoptosis Detection Kit I, BD Biosciences) binds to PS which is in turn linked to FITC hence can be detected through Flow cytometry as explained in material and methods. However, there was no significant change in the population of cells showing FITC labeling on treatment with BER at MIC_80_ as seen in Figure 6(b). For example, our data show that the percentage of gated cells labeled with Annexin V-FITC did not change significantly as it ranged between 0.67% in WT strain to 0.86% in cells treated with BER. This excluded the possibility that BER at MIC_80_ does not trigger apoptosis in *C. albicans* cells.

## Discussion

**Figure 7:**
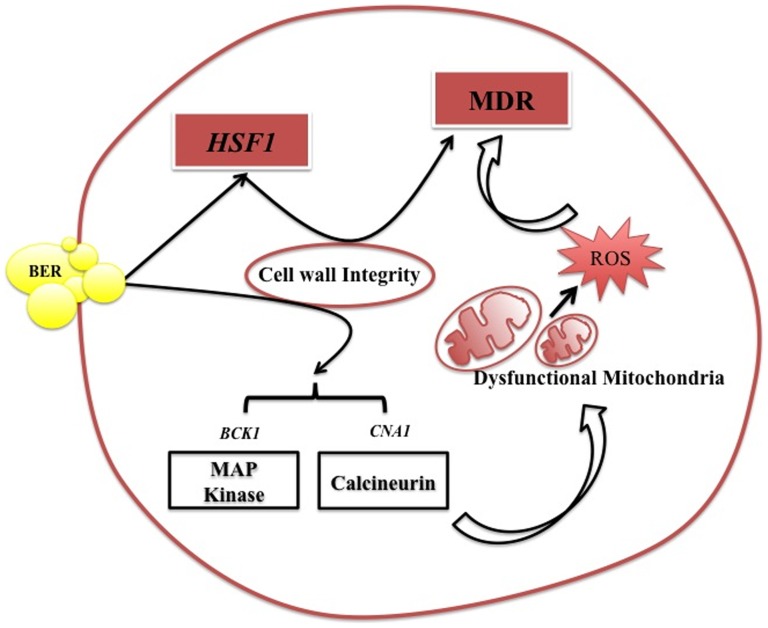
Model depicting pathways affected by BER treatment in *C. albicans*.

In this study, we show that the plant alkaloid BER displays potential anti-Candida activity which has been earlier reported to be non-toxic to humans [26] Our data supports that *HSF1*, a transcription regulator that is an essential gene involved in thermal adaptation of Candida cells, impacts BER susceptibility of *C. albicans* cells. *HSF1* protects cells from thermal assault by activating the expression of HSPs (Heat Shock Proteins) that act as chaperones to rescue proteins from misfolding under duress [19]. Our observation suggests that *HSF1*, which not only impacts BER susceptibility, also influences susceptibility of Candida cells to different drugs with distinct targets. Thus *HSF1* mutant is also observed to be collaterally susceptible to other drugs (Figure 3(b)). However, the impact of BER and *HSF1* on drug susceptibility is manifested by independent pathways in Candida cells. Our conclusions are based upon the following supporting observations. The treatment of TF mutant strains of Candida cells with BER revealed that *HSF1* mutant was most susceptible to this antifungal compound. BER treatment affects the calcineurin pathway and CW integrity leading to dysfunctional mitochondria and cell death. *HSF1* levels also impact CW integrity, which is apparent from the susceptibility of *HSF1* conditional mutants towards CW inhibitors and from disrupted CW visualized under TEM images. However, unlike BER treatment, the impact of *HSF1* on CW integrity was independent of calcineurin stress pathway. For example, neither *HSF1* levels could affect the expression of calcineurin pathway genes nor observed dysfunctional mitochondria and enhanced ROS levels following BER treatment were associated with *HSF1* phenotype. Although, *HSF1* levels potentiate the antifungal activity of BER but its influence in enhancing drug susceptibility adopts different regulatory circuitry as shown in Figure 7. Together, our results show that BER displays its antifungal potential through its ability to impair mitochondrial function, generation of ROS, targeting CW integrity pathway and also affecting heat shock transcription factor *HSF1*.

BER has been touted as potential anticancerous agent as it generates ROS and induces apoptosis in human prostrate cancer cells [38], however, our Annexin V labeling data rules out any induction of apoptosis in *C. albicans* following BER treatment (Figure 6(b)). Implication of BER in dysfunction of mitochondria in HSC-3 oral cancer cells has also been studied [39]. Another report shows that it is accumulated inside mitochondria, which results in the arrest of cell proliferation, mitochondrial fragmentation, along with depolarization and oxidative stress in K1735-M2 mouse melanoma cells [40]. Considering evidences from other systems, the possibility of other pathways, which may also govern BER susceptibility, could not be excluded.

Several major mechanisms of MDR have been identified. However, recent studies reveal newer regulatory pathways that govern the development of MDR in Candida cells. For instance, the ‘two component system’ composed of a membrane-bound, sensor histidine kinase (HK) protein, a cytoplasmic response regulator (RR) and an intermediate histidine phosphotransfer (HPt) protein. The two-component signal transduction pathways which help to adapt yeast cells to environmental stress is also shown to impact drug susceptibility of Candida cells [41]. The depletion of cellular iron pools leads to decrease resistance to several drugs involving ergosterol metabolism and calcineurin dependent core stress response pathways [42]. The role of mitochondria in MDR of *C. glabrata* is well documented [43]. Recently, electron transport defective null mutants of *goa1* and *ndh51* of *C. albicans* were shown to display increased susceptibility towards FLC [44]. Overwhelming emerging evidences also suggest an intricate relationship between membrane lipids and MDR wherein any compositional imbalance considerably influences drug susceptibility of Candida cells [45], [46]. Similarly, *CZF1*, a TF hitherto known to be responsible for white/opaque switching, was identified from whole genome RNA-Seq as another mediator the acquisition of MDR [47].

In the perspective of uncovering unconventional drugs that possess antifungal property, we had earlier shown that a chemical dye malachite green (MG), which is commonly used in fish industry as an antifungal, antiparasitic and antibacterial agent, could effectively kill *C. albicans* and non-albicans species [15]. MG owes its antifungal effect by shifting the metabolic flux towards fermentation, ROS generation leading to cell necrosis and is independent of known MDR mechanisms [15]. *UPC2* and *STP2* aid in tolerating MG stress of Candida cells [15]. In another study, natural polyphenol, curcumin (CUR) was shown to be cidal to *C. albicans* as well as to non-*albicans* species and display synergism when combined with known antifungals. CUR treatment induces ROS generation and an early apoptosis in *C. albicans* cells [11].

Together, the fungicidal effects of natural and synthetic compounds not only provide a platform to improve therapeutic strategies but also unravel novel regulatory circuitry that impacts MDR in Candida. This also indicates the flexibility of *C. albicans* cells to tolerate stress due to a variety of drugs with different targets by adopting different regulatory routes. Our results highlight that the development of tolerance towards antifungal drugs is a multifactorial phenomena, and the combat against MDR still remains a challenge.

## Supporting Information

File S1 **Supporting Information.** Figure S1 in File S1. Serial dilution assay showing MIC_50_ concentration for WT (BWP17) and *HSF1* mutant JMR044. Figure S2 in File S1. End point comparative RTPCR of genes involved in MDR in presence and absence of BER lane indicates 1: WT, 2: HSF1 TET/hsf1, 3: HSF1/hsf1, 4,5,6,: +Doxy, 7,8,9 :+BER, 10, 11, 12: +Doxy+Ber. Table S1 in File S1. List of strains used in the study. Table S2 in File S1. List of primers used in the study.
(DOC)
