# Supplementary material for: Molecular Mechanisms of Action of Herbal Antifungal Alkaloid Berberine, in Candida albicans
Source: PLoS One. 2014 Aug 8;9(8):e104554. doi: 10.1371/journal.pone.0104554 (PMC4126717; doi:10.1371/journal.pone.0104554)
Supplement: File S1 — Supporting Information. Figure S1 in File S1. Serial dilution assay showing MIC50 concentration for WT (BWP17) and HSF1 mutant JMR044. Figure S2 in File S1. End point comparative RTPCR of genes involved in MDR in presence and absence of BER lane indicates 1: WT, 2: HSF1 TET/hsf1, 3: HSF1/hsf1, 4,5,6,: +Doxy, 7,8,9 :+BER, 10, 11, 12: +Doxy+Ber. Table S1 in File S1. List of strains used in the study. Table S2 in File S1. List of primers used in the study. (DOC) [file pone.0104554.s001.doc]

**Supporting Information:**

Figure S1

Serial dilution assay showing MIC_50_ concentration for WT (BWP17) and *HSF1* mutant JMR044


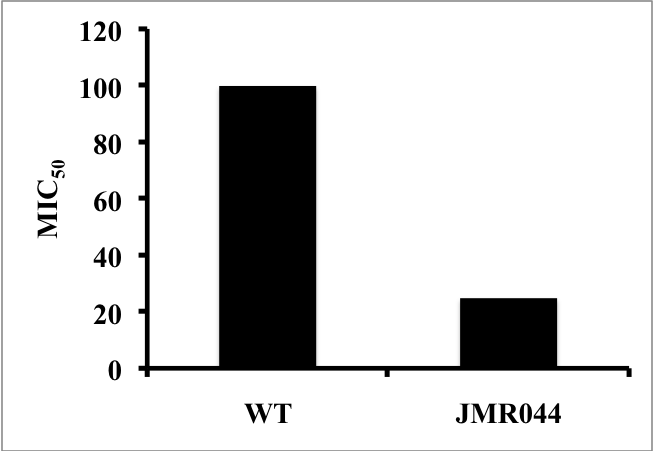


Figure S2

End point comparative RTPCR of genes involved in MDR in presence

and absence of BER lane indicates 1: WT, 2: HSF1 TET/hsf1, 3:

HSF1/hsf1, 4,5,6,: + Doxy, 7,8,9 :+ BER, 10, 11, 12: +Doxy+ Ber.


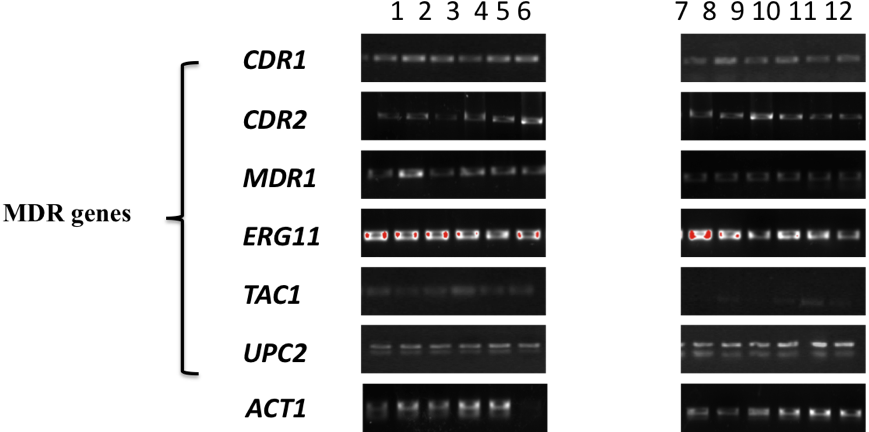


Table S1: List of strains used in the study:

| **Strains** | **Description** | **Reference** |
| --- | --- | --- |
| CAI4 *(WT)* | ura3/ura3 : : imm434 | 1 |
| Gu4 | Fluconazole susceptible isolate | 2 |
| Gu5 | Fluconazole resistant isolate overexpressing CDR1/CDR2 | 2 |
| F2 | Clinical isolate from patient F, Fluconazole sensitive | 3 |
| F5 | Clinical isolate from patient F, Fluconazole resistant, MDR1 overexpressing | 3 |
| DSY449 | Δcdr1::hisG/Δcdr1::hisG | 4 |
| DSY653 | Δcdr2::hisG-URA3-hisG/Δcdr2::hisG | 5 |
| SFLUM5 | mdr1::FRT/mdr1::FRT | 6 |
| ATCC 15239 | *C. utilis* | Ranbaxy Laboratories, India |
| ATCC 750 | *C. tropicalis* | Ranbaxy Laboratories, India |
| ATCC 90030 | *C. glabrata* | Ranbaxy Laboratories, India |
| ATCC 2512 | *C. kefyr* | Ranbaxy Laboratories, India |
| ATCC 6258 | *C. krusei* | Ranbaxy Laboratories, India |
| ATCC 33 | *C. dubliniensis* | Ranbaxy Laboratories, India |
| ATCC 22019 | *C. parapsilosis* | Ranbaxy Laboratories, India |
| MTCC 223 | *K. lactis* | National collection of Industrial Microorganism, India |
| DAY286 (*WT for JMR044*) | ura3Δ::λimm434/ura3Δ::λimm434 | 7 |
| Mutants made by URA blaster (11) | tf::hisG/tf::hisG-URA3-hisG | 8 |
| Mutants made by UAU transposition (182) | tf::ura3-ARG4-ura3/tf::URA3 | 8 |
| Mutants made by PCR from UAU cassette (24) | tf::ARG4/tf::URA3 | 8 |
| Mutants made by PCR from pFA plasmids (23) | RPS1/rps1::[CIp30] | 8 |
| THE1 *(WT for CLM61-1 and CLM62-1)* | ade2::hisG/ade2::hisG, ura3::l imm434/ura3::l imm434, ENO1/eno1::ENO1-tetR-ScHAP4AD-3XHA-ADE2 | 9 |
| CLM61-1 | ade2::hisG/ade2::hisG, ura3::l imm434/ura3::l imm434,  ENO1/eno1::ENO1-tetR-ScHAP4AD-3XHA-ADE2, hsf1::hisG/HSF1 | 10 |
| CLM62-1 | ade2::hisG/ade2::hisG, ura3::l imm434/ura3::l imm434, ENO1/eno1::ENO1-tetR-ScHAP4AD-3XHA-ADE2, hsf1::hisG/URA3-tetp-HSF1 | 10 |

Table S2: List of primers used in the study

| ***Gene name*** | ***Primer sequence*** |
| --- | --- |
| *HSF1 RTF*  *HSF1 RTR* | *GAATGGGCACGGATTTGCTG*  *ACGTCTTGAATGTGCTCGGT* |
| *ACT1RTF*  *ACT1 RTR* | *GGGTAGGGTGGGAAAACTTCA*  *TTGAAACCACTGCCGACAGA* |
| *CDR1 RTF*  *CDR1 RTR* | *AAGAGAACCATTACCAGG*  *AGGAATCGACGGATCAC* |
| *MDR1 RTF*  *MDR1 RTR* | *GGCGGATTTACTCCTGATACAACTC*  *GCGACGGGCTGTTGAGTAAACTAT* |
| *BCK1RTF*  *BCK1RTR* | *CCACACTGACGGGTTCGACA*  *CGATCACCTGGTGGTGGCGG* |
| *MCK1RTF*  *MCK1RTR* | *CCCGACGAAAATGCTGG*  *CGGGTTGGCGTCAGGGA* |
| *HSP90RTF*  *HSP90RTR* | *ACTTGTTGATGCTCCAGCTGCCA*  *CCAGCTGGTTCGTCAGTTGAGGC* |
| *CMP1RTF*  *CMP1RTR* | *ACTTGGTCATTACCGTTTGTGGGTG*  *CCTTCTCTTCTTCGTTGGCTCT* |
| *CNB1RTF*  *CNB1RTR* | *ATGGGGGCTAACGCAAGTATTCTTG*  *TCAGAACATATTTAATGTCAAAGTG* |
| *CRZ1RTF*  *CRZ1RTR* | *CAGGATGATGGGTCACAGC*  *ACAGTAGGTGCCGGTGGAGGT* |

References:

1. Fonzi WA, Irwin MY. Isogenic strain construction and gene mapping in Candida albicans. *Genetics* 1993; **134**:717-728.

2. Franz R, Ruhnke M, Morschhauser J. Molecular aspects of fluconazole resistance development in Candida albicans. *Mycoses* 1999; **42:**453-458.

3. Franz R, Kelly SL, Lamb DC *et al.* Multiple molecular mechanisms contribute to a stepwise development of fluconazole resistance in clinical *Candida albicans* strains. *Antimicrob.Agents Chemother* 1998; **42**:3065-3072.

4. Sanglard D, Calabrese D, Ischer F *et al*. Isolation of *Candida albicans* genes conferring resistance to azole antifungal agents. 1996. San Diego, USA. Ref Type: Conference Proceeding.

5.Sanglard D, Ischer F, Monod M *et al.* Cloning of *Candida albicans* genes conferring resistance to azole antifungal agents: Characterisation of *CDR2*, a new multidrug ABC transporter gene. *Microbiology* 1997; **143**:405-416.

6 Morschhauser J. 2002. The genetic basis of fluconazole resistance development in *Candida albicans*. *Biochem.Biophys.Acta* 2002; **1587**:240-248.

7. Davis DA, Bruno VM, Loza L *et al.* Candida albicans Mds3p, a conserved regulator of pH responses and virulence identified through insertional mutagenesis. *Genetics* 2002; **162**:1573-1581.

8. Dhamgaye S, Devaux F, Manoharlal R, *et al.* In Vitro Effect of Malachite Green on Candida albicans Involves Multiple Pathways and Transcriptional Regulators UPC2 and STP2. *Antimicrob Agents Chemother* 2012; **56**: 495–506.

9. Nakayama H, Moi T, Nagahashi S *et al.* Tetracycline-Regulatable System To Tightly Control Gene Expression in the Pathogenic Fungus Candida albicans. *Infect Immun* 2000; **68**: 6712–6719.

10. Nicholls S, Leach MD, Priest CL *et al.* Role of the heat shock transcription factor, Hsf1, in a major fungal pathogen that is obligately associated with warm-blooded animals. *Mol Microbiol* 2009; **74**: 844–861.
